# Physiological and oxidative status of soybean seedlings exposed to short term treatment with polystyrene nanoparticles

**DOI:** 10.1007/s10646-024-02833-0

**Published:** 2024-11-20

**Authors:** Michał Urbański, Burak Mete Yiğit, Anna Ekner-Grzyb, Jagna Chmielowska-Bąk

**Affiliations:** 1 Department of Plant Ecophysiology, Faculty of Biology, Institute of Experimental Biology, Adam Mickiewicz University, Poznań, Poland; 2 Department of Molecular Biology and Genetics, Gebze Technical University, Kocaeli, Turkey; 3 Department of Cell Biology, Faculty of Biology, Institute of Experimental Biology, Adam Mickiewicz University, Poznań, Poland

**Keywords:** Plastic, Polystyrene, Oxidative stress, *Glycine max*

## Abstract

Plastic is widely used worldwide due to its durability and relatively low production costs. However, its durability also has significant drawbacks - plastic is a slowly degrading material and greatly contributes to the environmental pollution. Increasing body of evidence shows that contamination of the environment with plastic negatively affects plants and other living organisms. The aim of present research was to determine whether short-term exposure to polystyrene nanoparticles (PSNP) has toxic effect on soybean seedlings (*Glycine max* L). In the first stage of the research, the effect of two hour long incubation in PSNP solutions (10 and 100 mgl^−1^) on the germination of soybean seeds was determined. In the second part of the study, the potential cytotoxic effect of PSNP on young seedlings was measured. The results indicate that incubation in PSNP solutions inhibits the germination of soybean seeds by approx. 10% (at *p* = 0.05). However, this effect was only observed after 48 and 72 h of germination and by lower PSNP concentrations, 10 mgl^−1^. In turn, in young soybean seedlings exposure to PSNP had no effect on growth, cell viability or oxidative status by *p* = 0.05. The results indicate that germination is a PSNP-sensitive process. In turn, already germinated seedlings are relatively resistant to the short-term exposure to this stressor.

## Introduction

As one of the most widely cultivated plant species worldwide, soybean holds crucial place in agriculture. According to FAO, in 2022 over 340 tons of soya beans were produced worldwide (FAOSTAT, https://www.fao.org/faostat, date of access: 30.10.2024). Soybean constitutes a crucial component of traditional cuisine in Asian countries and, since 20th century, is gaining increased significance as food product also in Western countries. It is valued mostly for its high proteins content reaching 46% in the beans. The high quantity and quality of proteins makes soya beans a good substitute for meat products e.g. in vegetarian and vegan diets. In addition, this crop is rich in unsaturated fatty acids exerting protective effects on cardiovascular system. Moreover, this plant produces some specific phytometabolites such as isoflavones, which are considered antioxidant and potentially anti-cancer compounds (Rizzo and Baroni 2018; Singer et al. 2023). The growth and yield of crops, including soybean, can be limited by stress conditions such as contamination of the environment (Oyebamiji et al. 2024). Among contaminants plastic is a relatively new factor - its large-scale production started in the middle of the 20^th^ century. Soon, it was noticed that due to its massive production and durability it can accumulate in significant amounts in the environment. In the time period 1950–2015 plastic production was estimated at 8300 million metric tons. Noteworthy, the majority (4800 million metric tons) was discarded into the environment (Geyer et al. 2017).

Plastic accumulated in the environment can be absorbed by various types of organisms including microorganisms, animals and plants. The exact impact of plastic particles on organisms is a relatively new topic of research. However, increasing number of studies evidence its toxicity (Okoffo et al. 2021). In some cases, exposure to plastic results in severe defects. For examples, in sea birds a new plastic dependent disease was described – plasticosis, which is associated with extensive formation of scar tissue (Charlton-Howard et al. 2023). Exposure to plastic poses health risks also for humans. Plastic particles can be taken up by through various elements of food chain e.g. bottled water, seafood, crop plants and sugar. In vitro assays carried out on human cell lines evidenced the cytotoxicic effects of plastic particles. In addition, studies on model animals indicate their potential negative impact on cardiovascular, nervous, immunological, digestive, respiratory and reproductive systems (Boriello et al. 2023, Khan and Jia 2023). In plants, exposure to plastic results in general in hampered growth, development of the symptoms of oxidative stress, disturbances in mineral homeostasis, alterations in metabolisms and damage of genetic material (reviewed in Ekner-Grzyb et al. 2022). However, it should be noted that some studies show no effect or even plastic-dependent stimulation of plants growth (Lian et al. 2020, Li et al. 2023).

In recent years, a significant increase in the number of publications on plastic impact on plants is observed. However, majority of the studies concern long term treatment times, while there is still limited number of reports on short term exposure. In addition, the influence of nanoplastic particles (NP) on the process of germination and growth of young seedlings is not elucidated in depth. The objective of present study was elucidation of the effects of exposure to polystyrene nanoparticles (PSNP) at two concentrations (10 and 100 mgl^−1^) on the process of soybean germination and the growth of young soybean seedlings. In the first part of the research, the germination rate was analyzed in seeds imbibed at PSNP solutions. The second part of the study, focused on the cytotoxic effects of PSNP on young seedlings, assessed by the potential changes on growth, cell viability and general oxidative status.

## Materials and methods

### Nanoplastic particles

In the study commercially available polystyrene nanoparticles (PSNP) of 100 nm of diameter with firefly fluorescent red dye (542 nm excitation/612 nm emission) were used (Thermo Fischer Scientific, R100B). The particles were dissolved in distilled water to appropriate concentrations (10 or 100 mgl^−1^) and were subjected to sonication before each application to prevent formation of aggregates.

### Cultivation and treatment procedures

For evaluation of the germination rate, soybean seeds were surface sterilized with 70% ethanol for 5 min and with 1% hypochlorite for 10 min, washed thoroughly and imbibed for 2 h in distilled water (control) or in the solution of PSNP at the concentration 10 or 100 mgl^−1^. For each treatment 50 seeds of similar size showing no sights of discoloration or seed coat damage were selected. After the imbibition, the seeds were washed thoroughly and places on glass Petri dishes (30 cm of diameter) lined with two layers of lignin and one layer of blotting paper. The seeds were watered with 30 ml of tap water and places in growing chamber, in the dark at 21–22 °C.

For the experiments on germinated seedlings, the seeds were sterilized as described above, washed under running water for 30 min and imbibed in tap water for 2–3 h. Then, the seeds were transferred to plastic trays lined with two layers of moistened lignin and one layer of blotting paper. The trays were covered with aluminum foil and placed in growing chamber, in the dark at 21–22 °C. After 3-days of germination the seedlings with similar root length were transferred to glass Petri dishes with two layers of blotting paper, wherein the upper layer had cut off wholes. The roots were placed in the wholes, in between the two layers of blotting paper. The seedlings were treated with 5 ml of distilled water (control) or 5 ml of PSNP at the concentration 10 or 100 mgl^−1^. The seedlings were transferred for 48 h to growing chamber (dark, 21–22 °C).

### Assessment of the germination rate

The number of germinated seedlings (with emerged radical) was counted after 24, 48 and 72 h and presented as %. The measurements were carried out in 4 independent repetitions and are expressed as mean ± SE.

### Detection of polystyrene nanoparticles in the roots

The microscopic observations were carried out on root sections of fresh seedlings treated with water (control) or PSNP for 48 h. The roots were thoroughly washed with distilled water, the sections were cut using razor and observed under Axiovert 200 M fluorescent microscope (Zeiss) with 546/607 nm excitation/emission light wave lengths. The photographs were taken on 5x magnitude using AxioCam MRC5 camera (Zeiss). The observations were made in three independent biological repetitions each consisting of approximately 7 cross sections derived from different roots. The intensity of fluorescence signal was quantified in seven sections from each experimental variant by the means of ImageJ software (Schneider et al. 2012). The results of fluorescence intensity are presented as mean ± SE.

### Evaluation of the growth and cytotoxic effects of PSNP

The roots length was measured using ruler, after 48 h of treatment. The results are mean ± SE of three independent experiments each comprising at least 20 seedlings. The cytotoxicity was evaluated by the Evans Blue uptake (Baker and Mock 1994). Approximately 200 mg of roots were cut off on ice and incubated in 0.25% Evans Blue (Sigma-Aldrich E2129) solution for 20 min. Thereafter, the roots were washed twice with distilled water and homogenized in de-staining buffer (1% SDS in 1:1 ethanol: distilled water) using TissueLyser II (2 min, 30 rounds/s). The samples were incubated 15 min at 50 °C, centrifuged (15 min, 12 000 rcf) and the absorbance of the supernatant was measured by λ = 600 nm on Synergy LX microplate reader (BioTek). The results were calculated per 1 g of FW and expressed as means ± SD. The procedure was carried out in 5 independent repetitions.

### Quantification of reduced (GSH) and oxidized (GSSG) glutathione

The GSH and GSSG level was evaluated by the means of Glutathione Assay Kit (Cayman 703002) according to the provided protocol and as described in Venkatesan et al. 2019. Briefly, approximately 200 mg of frozen roots were homogenized in MES buffer using TissueLyser II (2 min, 30 rounds/s). Then, the samples were centrifuged (10 min, 10,000 rcf, 4 °C) and the supernatant was deproteinated by 5 min incubation in 2.5% metaphosphorc acid (MPA, Sigma-Aldrich 239275) at RT and addition of 50 μl of 4 M triethanolamine (TEAM, Sigma-Aldrich T58300). Then the samples were divided into two parts – one part was used for GSH quantification and the other part was supplemented with 10 μl of 1 M 2-vinylpyridine (Sigma-Aldrich 13229-2), incubated 60 min at RT and used for GSSG measurements. For the GSH and GSSG quantification 50 μl of samples or standards were added to the microplate wells and were supplemented with 150 μl of Assay Cocktail. The absorbance was measured by 410 nm on Synergy LX microplate reader (BioTek) after 30 min of incubation in the dark. The results are means of four independent experimental repetitions ±SE.

### Assessment of lipid peroxidation

Lipid peroxidation was assessed based on changes in the level of thiobarbituric acid reactive substances (TBARS) (Dhindsa et al. 1981). Approximately 200 mg of roots were cut off on ice and homogenized in 10% trichloroacetic acids (TCA, Sigma-Aldrich T0699) using TissueLyser II (2 min, 30 rounds/s). The samples were centrifuged (12,000 rcf, 10 min) and 1 ml of supernatant was transferred to glass tubes and supplemented with 0.05% thiobarbituric acid (TBA, Sigma-Aldrich T5500). The reactive mixture was incubated for 30 min at 95 °C, cooled on ice and the absorbance was measured on Synergy LX microplate reader (BioTek) by λ = 532 and λ = 600 nm. The results by λ = 532 were corrected against unspecific absorbance at λ = 600 nm, calculated per one gram of fresh weight and divided by the extinction coefficient of 155 mM^−1^cm^−1^.

### Data availability statement and statistical analysis

The raw and curated data is available in the Zenodo repository: 10.5281/zenodo.13222443.

The one-way ANOVA was applied to assess the significant differences between PSNP treatments in relation to the control, using XL Miner Analysis ToolPak (Microsoft). The homogeneity of variances was carried out using Levene’s test calculator (https://www.statskingdom.com/). The statistically significant differences are marked with an asterisk (*).

## Results

**Fig. 1:**
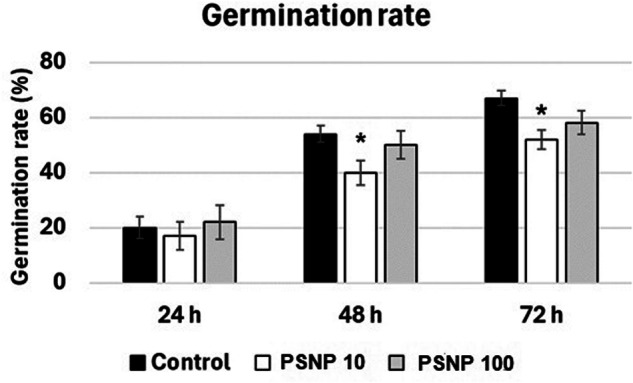
The impact of imbibition in water or PSNP at the concentration 10 (PSNP10) or 100 mgl^−1^ (NSPS100) on the germination rate of soybean seeds

The first stage of the research aimed to evaluate the impact of PSNP on the germination process. In general, a lower percentage of germination was observed in the case of seeds imbibed for 2 h in PSNP solution (Fig. 1). This result was statistically significant on *p* = 0.05 only by the seeds imbibed in 10 mg/l PSNP after 48 and 72 h of germination.

**Fig. 2:**
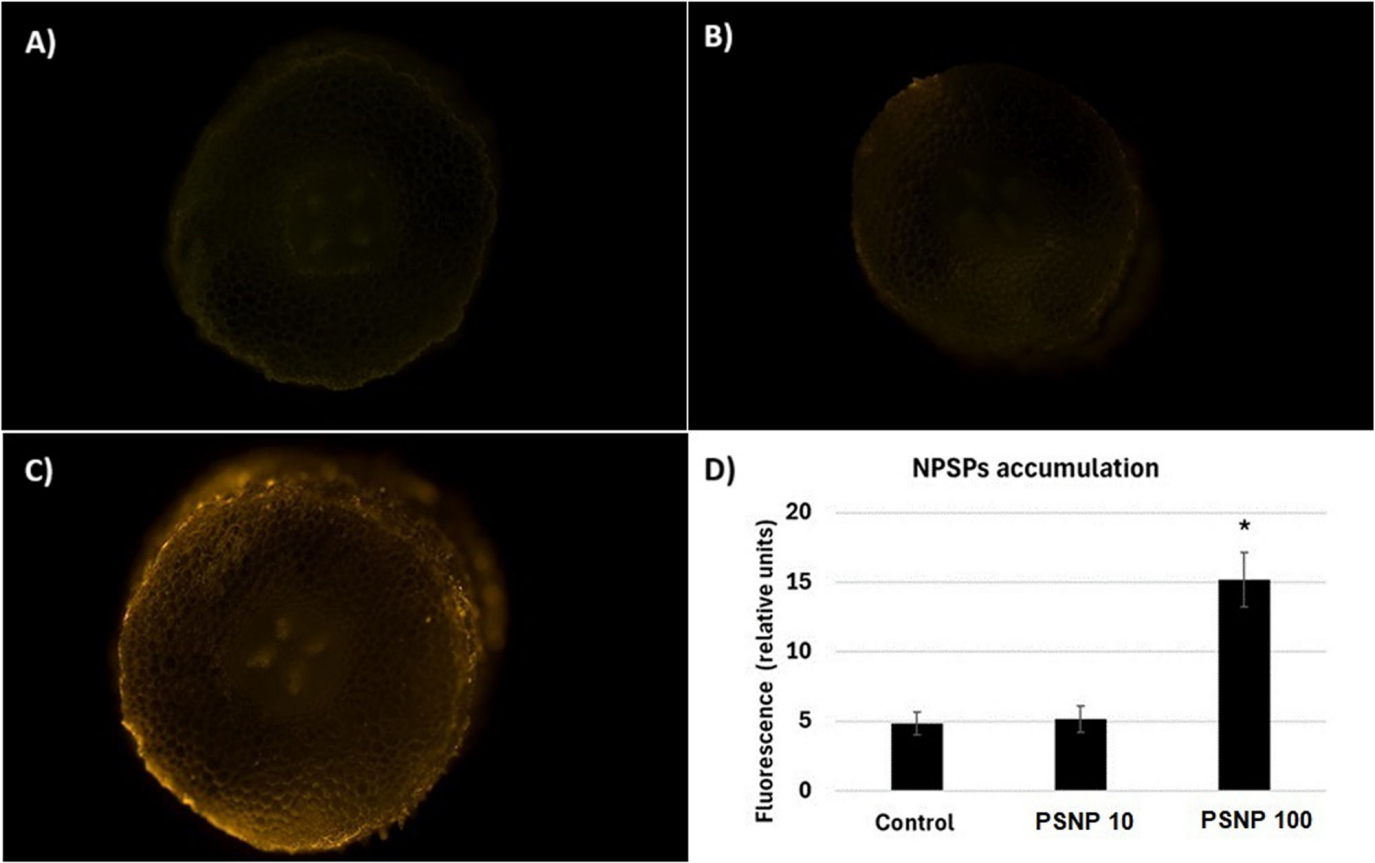
Detection of PSNPs in the control roots of soybean seedlings (Control, **A**) or in the roots of seedlings exposed to PSNPs at the concentration 10 (PSNP10, **B**) and 100 mgl^−1^ (PSNP100, **C**) for 48 h, densitometric measurements of fluorescence intensity in roots sections of all experimental variants (**D**)

In the next stage of the research the influence of PSNP was assessed on already germinated, 3 days old seedlings. Application of PSNP with fluorescent dye enabled their detection in plant tissues. In the roots of control seedlings, faint green autofluorescence was observed in the root cross section (Fig. 2A). The image of the root cross section of seedlings exposed for 48 h to 10 mgl^−1^ PSNP did no differ significantly from the image of control seedlings (Fig. 2B). On the other hand, strong yellow-orange signal was detected in the root cross sections of the seedlings treated with 100 mgl^−1^. The signal was located mainly by the rhizodermis and in vascular tissues (Fig. 2C). The observations were confirmed by the measurements of fluorescence intensity showing significantly stronger signal in the cross section of seedling exposed to 100 mgl^−1^ PSNP (Fig. 2D).

**Fig. 3:**
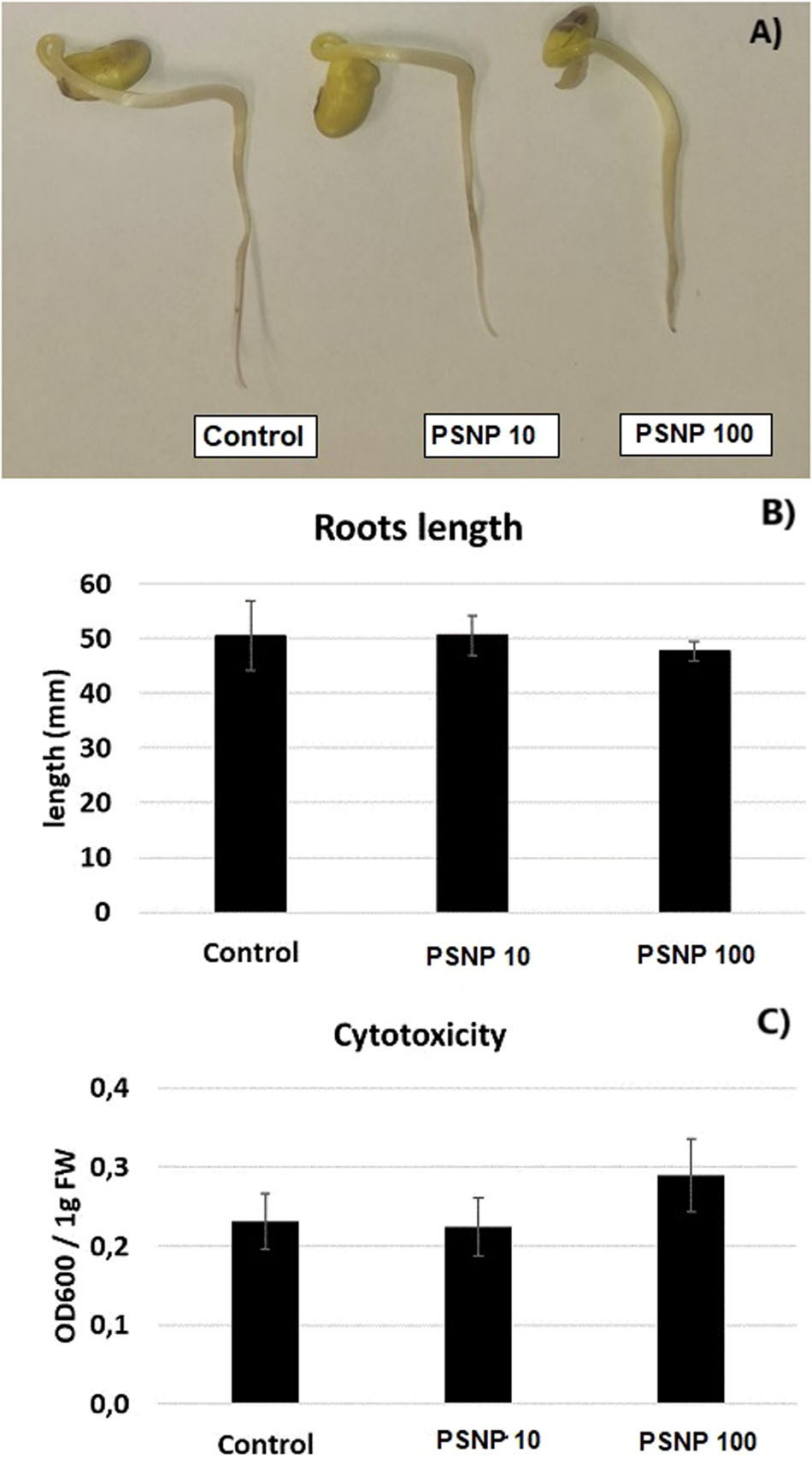
Assessment of the PSNP cytotoxic effects - the morphology (**A**), roots length (**B**) and cell mortality (**C**) of control roots of soybean seedlings (Control) or in the roots of seedlings exposed to PSNP at the concentration 10 (PSNP) and 100 mgl^−1^ (PSNP100) for 48 h (**A**–**C**)

The short-term treatment with PSNP did not have visible effects on seedlings’ morphology (Fig. 3A). In addition, no differences were noted in the case of roots growth (Fig. 3B) or cell mortality evaluated by the Evans Blue uptake (Fig. 3C).

**Fig. 4:**
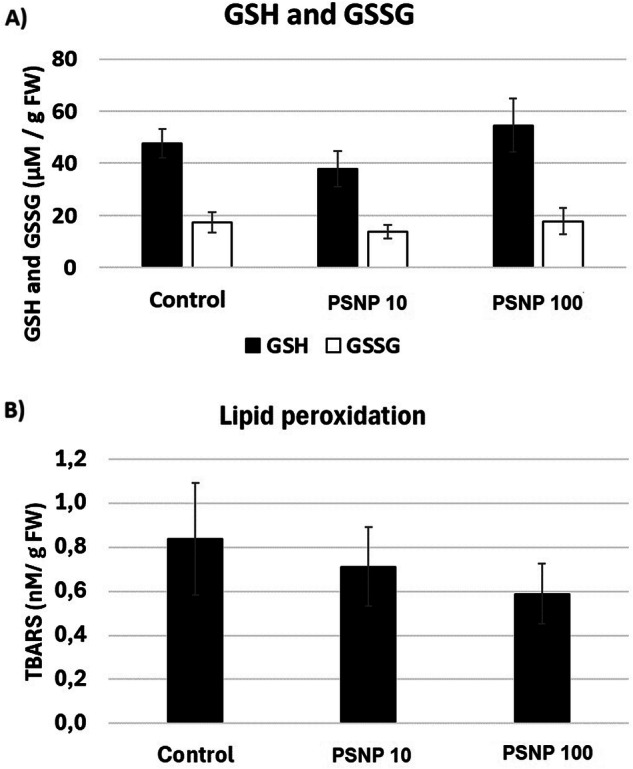
The oxidative status of soybean seedlings – the level of reduced and oxidized glutathione (**A**) and lipid peroxidation (**B**) in the roots of control soybean seedlings (Control) or in the roots of seedlings exposed to PSNP at the concentration 10 (PSNP10) and 100 mgl^−1^ (PSNP100) for 48 h

Treatment with PSNP also had no impact on the level of reduced and oxidized glutathione (Fig. 4A). The level of GSH was approximately threefold higher than the level of GSSG in all experimental variants, indicating no changes in the general oxidative status. Similarly, no significant changes were observed in the case of TBARS levels reflecting lipid peroxidation (Fig. 4B).

## Discussion

Germination is one of the crucial processes in plants life cycle and is tightly regulated by internal and external factors. This process is also modulated by stresses such as pollution (Wang et al. 2024). Exposure to plastic resulted in hampered germination in tomato (*Solanum lycopersicum* L.) (Lakshmikanthan and Chandrasekaran 2022). In rapeseed plants (*Brassica napus* L.), NP concentration exceeding 50 mgl^−1^ led to decrease in germination rate and seeds vigor (Li et al. 2023). In rice, lower concentrations of NP caused inhibition of germination, while higher concentration had no effect (Spanó et al. 2022). Similarly, in the present study treatment with PSNP at the concentration 10 mgl^−1^ hampered germination, while higher concentrations (100 mgl^−1^) had no effect (Fig. 1). Thus, present results and literature data show that the effect of NP on the process of germination depends on various factors including plants species, NP type, concentration and treatment duration. The hampered germination might depend on the alterations in water uptake during the imbibition. However, we did not observe differences in water absorption reflected by lack of differences between seeds weight before and after the imbibition in any of the experimental variants (data not shown). Plastic particles could also modulate hormonal signaling or other signaling elements engaged in the regulation of germination. Indeed, transcriptomic analysis revealed that exposure to plastic particles induces changes in the expression of genes associated with phytohormones metabolism and signaling pathways (reviewed in Ren et al. 2024).

Application of NP conjugated with fluorescent dyes enables their detection in plant tissues. This method enabled localization of NP e.g. in cucumber seedlings (Li et al. 2021), chicory (Muccifora et al. 2022) and castor bean (Jiang et al. 2019). In the present study strong fluorescent signal was observed in roots cross sections of seedlings treated for 48 h with PSNP at the concentration 100 mgl^−1^ (Fig. 2). The signal was located by the rhizodermis and in vascular bundles. Its localization in vascular bundles suggests that PSNP are transported with water through xylem. This would stand in line with the studies implying that aquaporins are involved in the uptake of plastic particles (Maity et al. 2024). The signal observed by the rhizodermis might be associated with PSNP uptake and transport within the root. However, it could be also derived from PSNP bond to the root surface.

The majority of the studies evidence inhibitory effects of NP on plants growth (e.g. Giorgetti et al. 2020, Jiang et al. 2019, Lakshmikanthan and Chandrasekaran 2022, Li et al. 2021, Santini et al. 2023, Wu et al. 2023, Spanó et al. 2022, Zhou et al. 2021). In addition, NP induced accumulation of reactive oxygen species (ROS) and/or oxidative stress markers (reviewed in Ekner-Grzyb et al. 2022). In the present study, short term, 48 h long treatment with PSNP did not affect growth, cell viability or oxidative status in the roots of soybean seedlings (Figs. 3 and 4). No effect or even NP-dependent growth stimulation was already described in individual reports. For example, in rapeseed plants, roots growth was stimulated by lower plastic concentrations but inhibited at higher concentrations (Li et al. 2023). In wheat, PSNP induced shoots and roots biomass (Lian et al. 2020). It should be noted that the present study is focused on the early reactions. Thus, it is possible that toxicity symptoms would occur after longer treatment periods. Nonetheless, the results indicate that soybean germination is a sensitive process to PSNP action, while already germinated seedlings are relatively tolerant to this stressor. For comparison, treatment with another contaminant, Cd, in nearly identical experimental conditions and in significantly lower concentrations (25 mgl^−1^) caused reduction in roots growth, increase in cells’ mortality and enhanced lipid peroxidation (Holubek et al. 2020).

## Conclusions

The results of present study show that seeds imbibition in the solution of polystyrene nanoparticles (PSNP) leads to hampered germination. This effect is concentration dependent, observed by lower (10 mgl^−1^) but not higher (100 mgl^−1^) PSNP concentrations. On the other hand, already germinated seedlings are tolerant to short term PSNP treatment, as reflected by unchanged growth, cell viability or oxidative status. The results show that plants reaction to plastic is dependent on various factors including plant species and plastic type, size, concentration and treatment time. Further research is needed to get more complex insights into plants reaction to this stressor and for elucidation of tolerance/defense mechanisms and possible elaboration of food safety regulations and remediation strategies. The inhibitory effect of NP on the germination process would be an interesting topic for future research as alterations in germination could greatly affect crops productivity. On the other hand, so far this topic gained little attention and the exact mechanism of NP interference with the germination process is yet to be discovered.

## Data Availability

The raw and curated data is available in the Zenodo repository: 10.5281/zenodo.13222443.
